# Parental Transfer of the Antimicrobial Protein LBP/BPI Protects *Biomphalaria glabrata* Eggs against Oomycete Infections

**DOI:** 10.1371/journal.ppat.1003792

**Published:** 2013-12-19

**Authors:** Olga Lucia Baron, Pieter van West, Benoit Industri, Michel Ponchet, Géraldine Dubreuil, Benjamin Gourbal, Jean-Marc Reichhart, Christine Coustau

**Affiliations:** 1 Sophia Agrobiotech Institute, INRA-CNRS-UNS, Sophia Antipolis, France; 2 Institut de Biologie Moléculaire et Cellulaire, UPR9022 CNRS, Strasbourg, France; 3 Aberdeen Oomycete Laboratory, University of Aberdeen, Institute of Medical Sciences, Foresterhill, Aberdeen, United Kingdom; 4 Ecologie et Evolution des Interactions, UMR 5244 CNRS, Université de Perpignan Via Domitia, Perpignan, France; George Washington University School of Medicine and Health Sciences, United States of America

## Abstract

Vertebrate females transfer antibodies via the placenta, colostrum and milk or via the egg yolk to protect their immunologically immature offspring against pathogens. This evolutionarily important transfer of immunity is poorly documented in invertebrates and basic questions remain regarding the nature and extent of parental protection of offspring. In this study, we show that a lipopolysaccharide binding protein/bactericidal permeability increasing protein family member from the invertebrate *Biomphalaria glabrata* (BgLBP/BPI1) is massively loaded into the eggs of this freshwater snail. Native and recombinant proteins displayed conserved LPS-binding, antibacterial and membrane permeabilizing activities. A broad screening of various pathogens revealed a previously unknown biocidal activity of the protein against pathogenic water molds (oomycetes), which is conserved in human BPI. RNAi-dependent silencing of LBP/BPI in the parent snails resulted in a significant reduction of reproductive success and extensive death of eggs through oomycete infections. This work provides the first functional evidence that a LBP/BPI is involved in the parental immune protection of invertebrate offspring and reveals a novel and conserved biocidal activity for LBP/BPI family members.

## Author Summary

Vertebrate immune systems not only protect adult organisms against infections but also increase survival of offspring through parental transfer of innate and adaptive immune factors via the placenta, colostrum and milk or via the egg yolk. This maternal transfer of immunity is critical for species survival as embryos and neonates are immunologically immature and unable to fight off infections at early life stages. Parental immune protection is poorly documented in invertebrates and how the estimated 1.3 million of invertebrate species protect their eggs against pathogens remains an intriguing question. Here, we show that a fresh-water snail, *Biomphalaria glabrata* massively loads its eggs with a lipopolysaccharide binding protein/bactericidal permeability increasing protein (LBP/BPI) displaying expected antibacterial activities. Remarkably, this snail LBP/BPI also displayed a strong biocidal activity against water molds (oomycetes). This yet unsuspected activity is conserved in human BPI. Gene expression knock-down resulted in the reduction of snail reproductive success and massive death of eggs after water mold infections. This work reveals a novel and conserved biocidal activity for LBP/BPI family members and demonstrates that the snail LBP/BPI represents a major fitness-related protein transferred from parents to their clutches and protecting them from widespread and lethal oomycete infections.

## Introduction

The existence of complex immune systems implies that interactions with pathogens represent major selective forces shaping the evolution of animal and plant species [1]. Vertebrate immune systems not only protect the adult organism against infections but also increase reproductive success through parental transfer of innate and adaptive immune factors via the placenta, colostrum and milk or via the egg yolk [2]–[4]. This maternal transfer of immunity is critical for species survival as embryos and neonates are immunologically immature and unable to fight off infections at early life stages. Parental transfer of protection has also been found in invertebrates hosting mutualists and many vertically transmitted arthropod symbionts are able to protect offspring against specific infections [5], [6]. Despite the impressive advances recently made in characterizing invertebrate immune systems [7], [8], data on the nature of the symbiont-mediated or parentally transmitted protection across generations are scarce [9]–[11]. How the estimated 1.3 million of invertebrate species [12] protect their offspring against pathogens remains therefore an intriguing question.

The freshwater snail *Biomphalaria glabrata* is particularly well studied as it is the intermediate host of the human blood fluke *Schistosoma mansoni*, responsible for schistosomiasis affecting millions of people in developing countries [13]. *Biomphalaria* snails live in various resting water biotopes such as, ponds, marshes, irrigation channels or open sewer drains that are particularly rich in pathogenic organisms. Egg masses are laid on solid substrates under water where they remain for approximately a week before hatching [14]. In a proteomic study on the content of *B. glabrata* egg masses, 16 defense-related polypeptides were partially identified, among which a lipopolysaccharide binding protein/bactericidal permeability increasing protein (LBP/BPI) representing a major protein band [15]. LBP/BPIs are structurally related proteins belonging to the lipid transfer/binding protein (LT/LBP) family [16], which represent important components of the innate immune system against Gram-negative bacterial infections [17]. In mammals, LBPs and BPIs have been extensively studied due to their role in regulating transducing cellular signals from Lipopolysaccharide (LPS) [18], [19]. LBP functions as a carrier of LPS monomers onto CD14 and together with the TLR4-MD2 receptor complex, mediates the activation of monocytes and macrophages, which produce inflammatory mediators [20]. BPI is an antibacterial protein specifically active against Gram-negative bacteria that acts by damaging bacterial membranes [21]. BPI also enhances adaptive immune responses by promoting LPS uptake and presentation to dendritic cells [22]. Although these two proteins present similarities in sequence and activities, they exert different effects on interactions of the host with Gram-negative bacteria [23]. BPI neutralizes the inflammatory properties of LPS decreasing its uptake by LBP whereas LBP is an acute phase protein with LPS-dependent cell stimulatory activity [24], [25]. These antagonist functions efficiently regulate host response to bacterial invasion and allow the host immune system to return to its normal resting state.

The distinction between LBPs and BPIs has not been established in invertebrates. LBP/BPI family members have been reported only in a few invertebrate phyla such as annelids [26] and molluscs [27], [28]. To date, a single functional study of LBP/BPI has been performed, showing that the oyster *Crassostrea gigas* expresses a BPI-like protein endowed with the conserved LPS-binding and bacterial permeability increasing activity [27].

As *B. glabrata* snails apparently heavily invest in the production of LBP/BPI in their eggs [15], we investigated whether this protein showed the expected anti-bacterial activity and whether it could provide protection against other pathogens.

## Results

### BgLBP/BPI1 characterization

**Figure 1:**
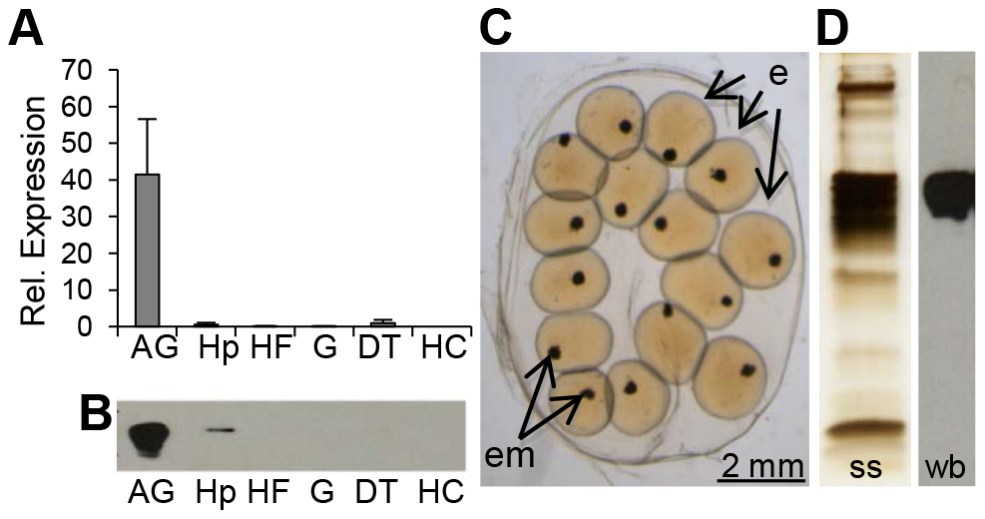
BgLBP/BPI1 is expressed in the albumen gland and is the major protein of *B. glabrata* egg masses. (A) Relative expression levels of BgLBP/BPI1 transcripts in the albumen gland (AG), hepatopancreas (Hp), Headfoot (HF), gonads (G), digestive tract (DT) and circulating hemocytes (HC) determined using real-time quantitative PCR (data from 3 independent experiments). Expression has been normalized to expression of S19 and EEF1-α reference genes. (B) Representative western blot showing the content in BgLBP/BPI1 protein in 15 µg total protein from the corresponding snail tissues (legend as in (A). (C) Typical live 2-days old *B. glabrata* egg mass with eggs (e) containing embryos (em) observed under a phase-contrast reversed microscope. (D) Total egg mass protein content as revealed by silver staining (ss) and egg mass BgLBP/BPI1 revealed by western blot (wb) using a custom-made antibody raised against two BgLBP/BPI1 peptides.

We first characterized the complete coding sequence of BgLBP/BPI1 (genbank accession number KC206037) from a partial transcript that we had previously identified in an albumen gland cDNA library [29], as this organ is known in gastropod snails to produce many components of the egg masses, among which the egg perivitelline fluid [30]. Interestingly, BLAST searches against non-redundant protein databases using the BLASTp program revealed that BgLBP/BPI1 corresponded to the “Developmentally regulated albumen gland protein” (partial sequence; genbank accession number AAB00448.1) previously identified as being over-expressed in *Schistosoma mansoni* resistant snails [31]. The sequence of BgLBP/BPI1 displayed the typical features of LBP/BPI family members, including a N-terminal LBP/BPI domain (pfam PF01273, Interpro IPR017942) containing conserved lysines involved in the interaction with LPS, a central proline-rich domain, and a C-terminal LBP/BPI domain (Figure S1) [17], [32]. Expression studies showed that the major site of expression for BgLBP/BPI1 was the albumen gland (Figure 1, A and B). We confirmed, using a specific antibody and western blot analysis followed by mass spectrometry, that the BgLBP/BPI1 gene product is the major protein found in egg masses of *B. glabrata* (Figure 1, C and D).

### *In vitro* biological activities

**Figure 2:**
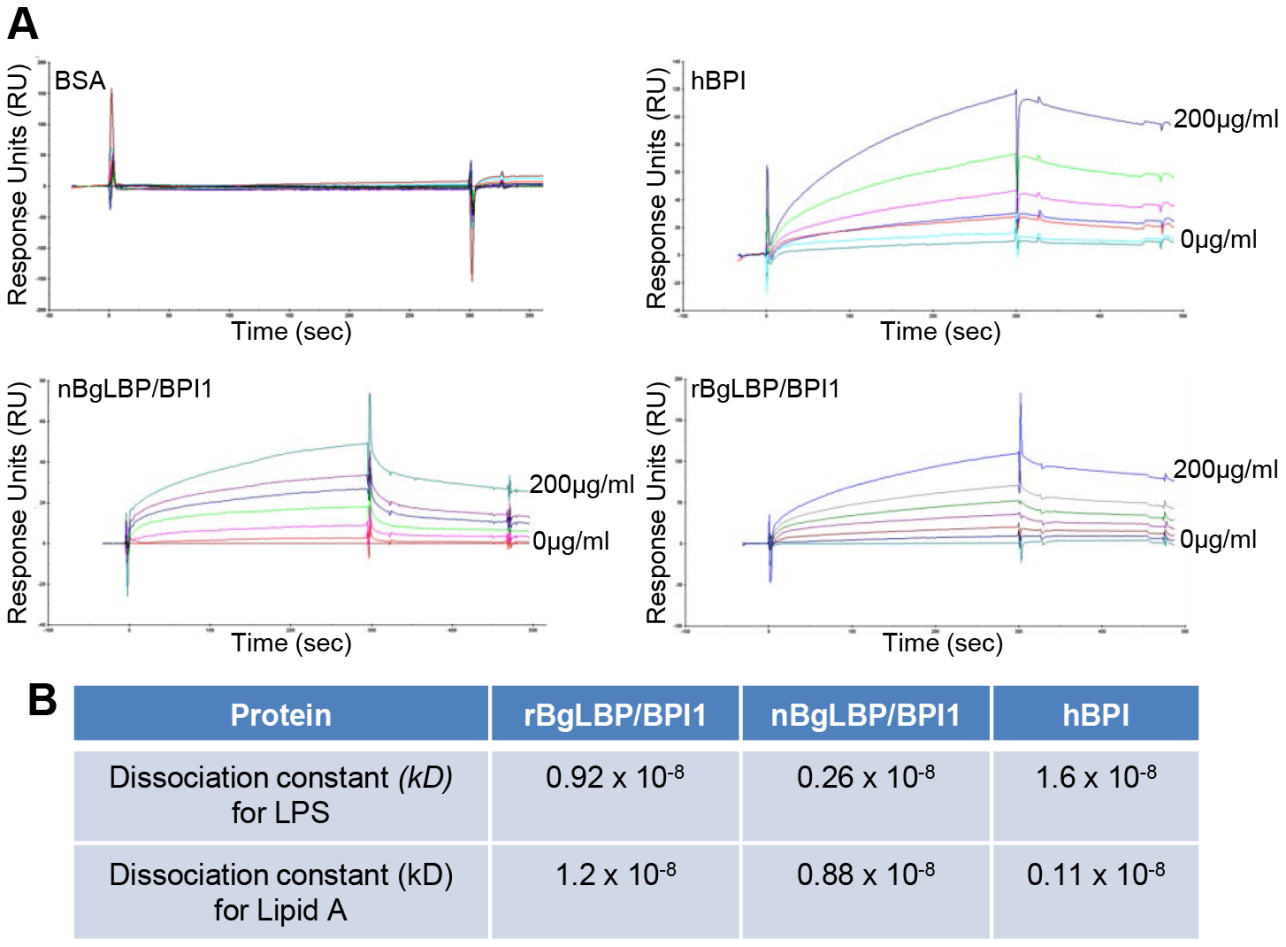
BgLBP/BPI1 bind to LPS and lipid A. (A) Representative plasmon resonance sensorgrams showing the interactions of BSA (negative control), hBPI (positive control), nBgLBP/BPI1 and rBgLBP/BPI1 with LPS. Purified proteins were immobilized on a CM5 sensor chip and serial LPS dilutions (5, 10, 25, 50, 100 and 200 µg/ml) were injected at a flow rate of 50 ul/min. The responses in RU were recorded as a function of time. Data were fitted to a 1∶1 Langmuir binding model. Similar sensorgrams were obtained with lipid A. (B) Dissociation constants (*kD*) calculated for each protein.

**Figure 3:**
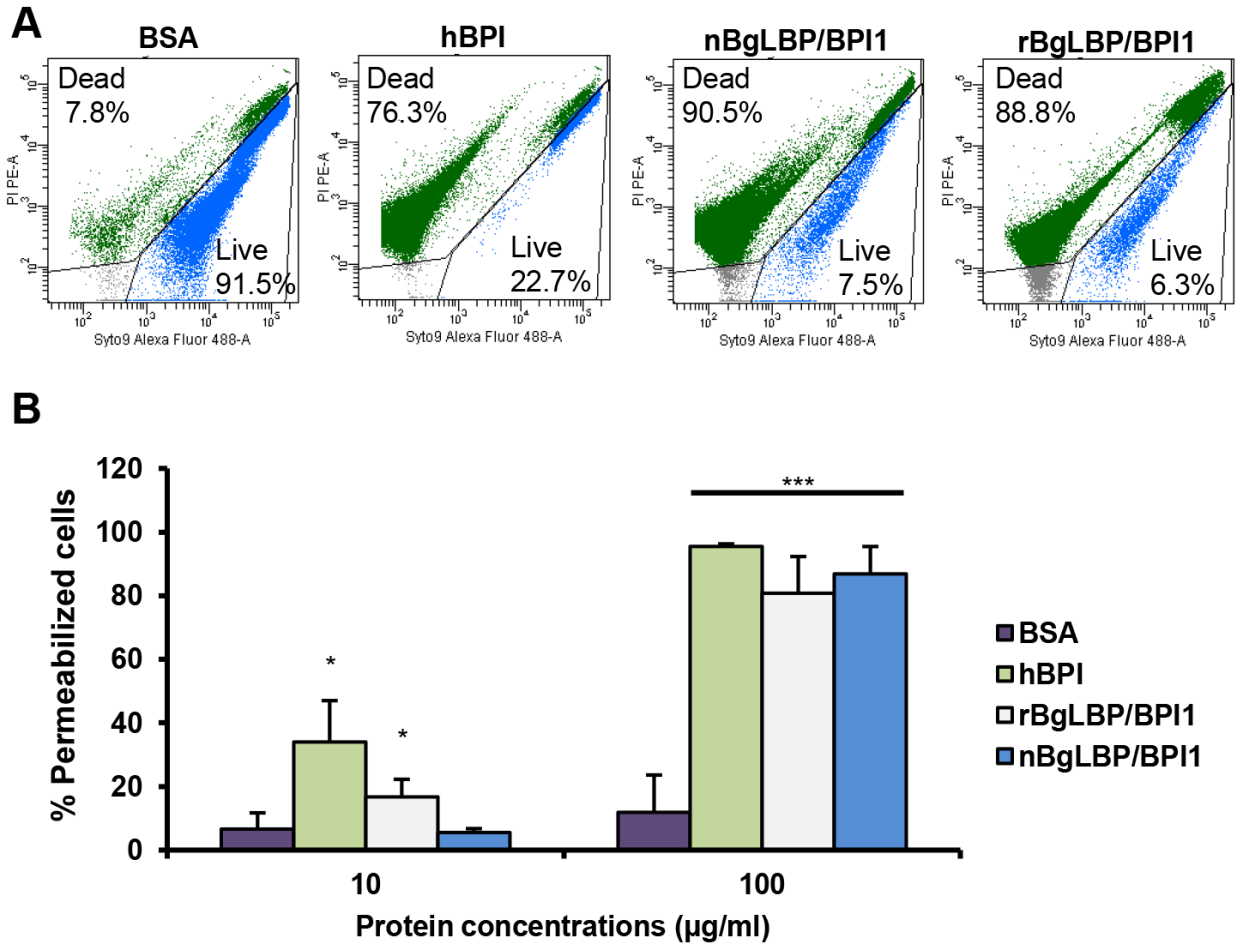
BgLBP/BPI1s permeabilize membranes and induce death of short LPS *E. coli*. (A) Representative flow cytometry profiles of *E. coli SBS363* cells exposed to BSA (negative control), hBPI (positive control), native (n) BgLBP/BPI1 and recombinant (r) BgLBP/BPI1 at a concentration of 100 µg/ml, for 1 h. Cells have been stained with the Baclight kit (SYTO 9 and PI) and a total of 60,000 events were recorded for each sample. (B) Quantification of the effect of the four proteins on *E. coli SBS363* cell death (as measured in (A)). Cell death has been measured by flow cytometry after 1 hour exposure to 10 or 100 µg/ml proteins. Results are the mean percentages of permeabilized cells ± SE of three independent experiments. Asterisks indicate significant differences with negative control (*p<0.05, ***p<0.001).

We purified the native BgLBP/BPI1 protein from fresh egg masses and estimated its physiologic concentration around 100 µg/ml of egg mass extract, representing 60% of the total protein dry weight. In order to control for trace contamination of the purified native protein by biologically active polypeptides, we produced a recombinant protein in *Drosophila* S2 cell culture and compared both the native and the recombinant proteins in our assays. Plasmon resonance analysis confirmed that both BgLBP/BPI1 bind LPS and the Lipid A region, which are common to all LPS's, with a range of affinity similar to that of human BPI protein, as shown by their dissociation constants (Figure 2) [33]. In addition, both proteins showed the typical membrane permeabilizing activity leading to bacterial death (Figure 3) that LBP/BPI proteins present toward short-LPS strains of *E. coli* [34], demonstrating that the biocidal activity of LBP/BPI proteins against Gram-negative bacteria is conserved in BgLBP/BPI1 [21], [35], [36].

**Figure 4:**
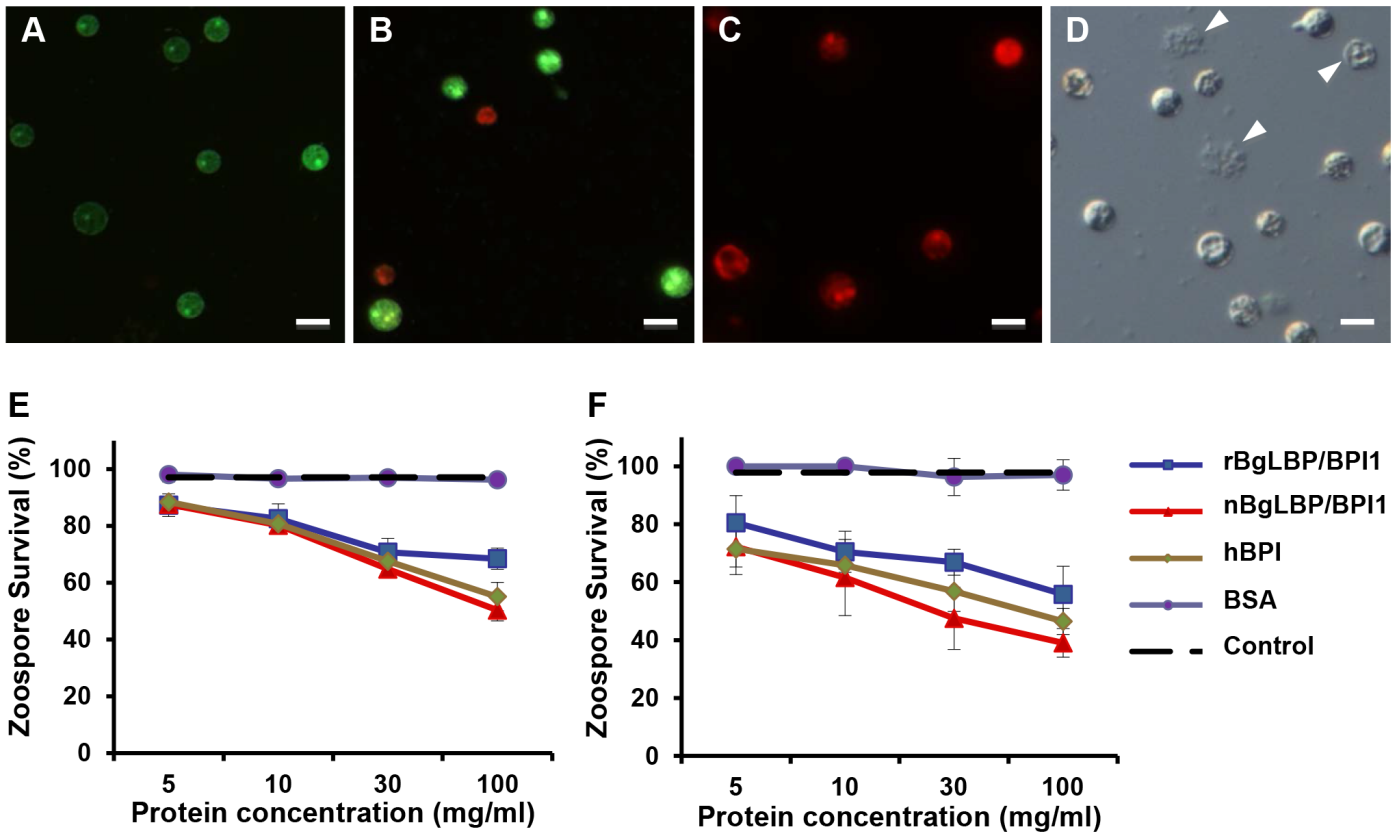
BgLBP/BPI1 and hBPI display anti-oomycete activity against *Saprolegnia parasitica* and *S. diclina*. (A–C) Pictures of live (green) or dead (red) *Saprolegnia* zoospores after exposure to BSA (A), or BgLBP/BPI proteins at 10 µg/ml (B) or 100 µg/ml (C). Zoospores have been stained by the live/dead cell assay (Abcam). Scale bar is 20 µm. (D) Phase contrast observation of zoospores exposed to 10 µg/ml BgLBP/BPI. Arrows show examples of dead cells or cell debris. (E, F) Survival rate of *Saprolegnia parasitica* (E) and *S. diclina* (F) after 30 min exposure to buffer alone (Control) or increasing concentrations of rBgLBP/BPI1, nBgLBP/BPI1, hBPI and BSA. Results are mean percentages (± SE) of three independent experiments.

**Figure 5:**
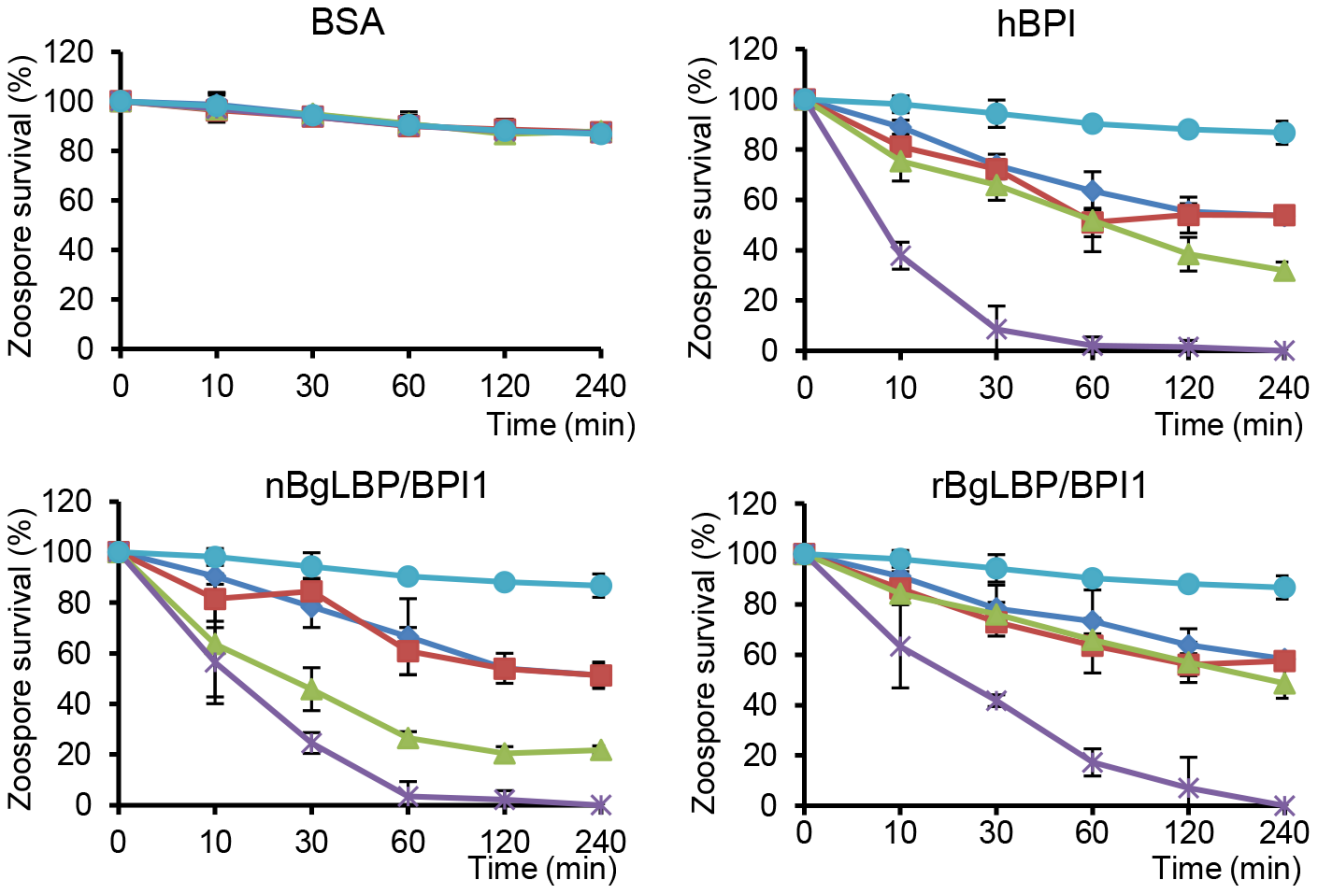
BgLBP/BPI1s and hBPI display anti-oomycete activity against *P. parasitica* zoospores. Survival rate of *P. parasitica* zoospores after exposure to BSA, hBPI, rBgLBP/BPI1 and nBgLBP/BPI1. Zoospores were incubated with proteins at 5 (dark blue), 10 (red), 30 (green) and 100 µg/ml (purple). Negative controls (light blue) are zoospores without treatment. Results are mean percentages (± SE) of three independent experiments.

Exposure of helminths (*S. mansoni*), Gram-positive bacteria (*Micrococcus luteus*, *Bacillus cereus*), Gram-negative bacteria (*Citrobacter freundii*, and *Pseudomonas aeruginosa*) and fungi (*Candida albicans* and *Saccharomyces cerevisiae*) to increasing concentrations of the recombinant BgLBP/BPI1 (up to the physiological concentration of 100 µg/ml) had no significant effect on the viability of the microorganisms (*P*>0.1, Figure S2 and S3). With the exception of helminths such as *S. mansoni*, information on natural pathogens of *B. glabrata* is scarce. Therefore we also wanted to investigate whether oomycetes are sensitive to BgLBP/BPI1. Oomycetes or water molds are a large group of eukaryotic microbes that can infect plants and animals and can cause devastating diseases in agriculture, aquaculture, and natural (aquatic) ecosystems [37], [38]. The motile zoospores (infective stage) and cysts (germinal stage) of both *Saprolegnia parasitica* and *Saprolegnia diclina*, two well-known pathogens of fresh water fish and their eggs [38], were exposed to increasing concentrations of BgLBP/BPI1 proteins for 30 min. No effect was observed on *Saprolegnia* cysts (not shown), but a strong biocidal activity was observed on the infective stage of these pathogens at all protein concentrations (Figure 4). The viability of *S. parasitica* or *S. diclina* zoospores was significantly reduced to 50.3% and 68.4% or 55.8% and 39% with 100 µg/ml of native BgLBP/BPI1 and recombinant BgLBP/BPI1, respectively (Figure 4). Interestingly, human BPI also strongly decreased the viability of both oomycete species (Figure 4), revealing a yet unsuspected activity of this well-studied human immune protein [39]. To better assess the LBP/BPI-dependent anti-oomycete activity, we also tested a plant pathogenic species, *Phytophthora parasitica* that has the ability to form biofilms [40] like Gram-negative bacteria. *Biomphalaria* proteins were tested on the three stages of biofilm formation; zoospores, cysts and microcolonies (Figure S4A). Similarly to the *Saprolegnia* species, only zoospores were affected by the BgLBP/BPI proteins. Both the snail and the human LBP/BPI proteins showed a strong biocidal activity of zoospores from *P. parasitica* in a dose dependent manner (Figure 5 and S4B). The effect of LBP/BPIs was observed as early as 10 min and resulted in 100% zoospore mortality after one or two hours of exposure to physiological concentrations of 100 µg/ml LBP/BPI proteins (Figure 5), confirming the strong biocidal activity of LBP/BPIs against oomycete zoospores.

### Demonstration of the anti-oomycete activity *in vivo*

**Figure 6:**
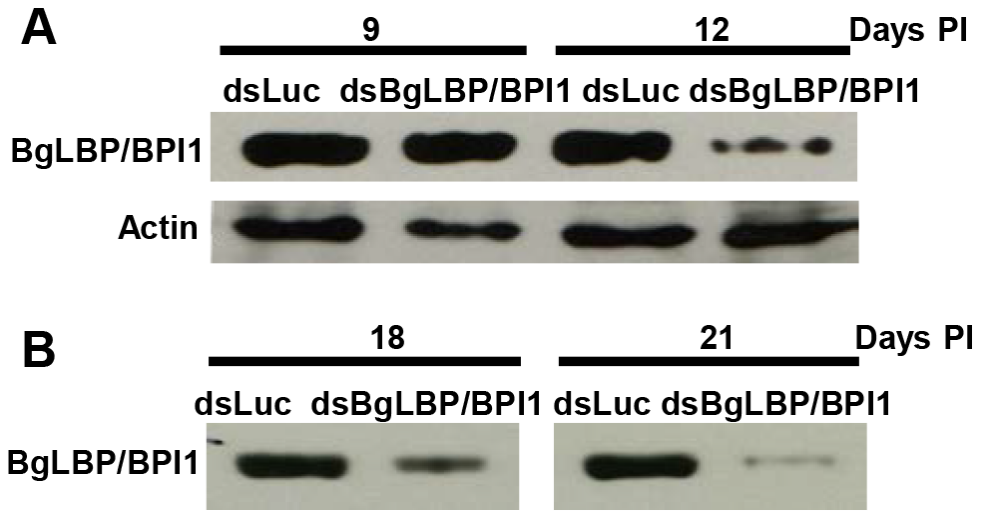
Injection of BgLBP/BPI1 dsRNA results in a substantial decrease in BgLBP/BPI1 protein in the albumen gland and in egg masses. (A) Western blots of albumen glands from snails at 9 days and 12 days post injection (Days PI) of dsRNA of luciferase (non-relevant dsRNA) (dsLuc) or BgLBP/BPI1 (dsBgLBP/BPI1). Actin was used as an endogenous control for protein loads. (B) Western blots of egg masses laid by snails at 18 and 21 days post injection of dsRNAs. Note that control for actin or total protein contents were not appropriate for egg masses and that protein loads have been standardized towards egg mass dry weights. Western Blots were performed using a custom-made antibody raised against two BgLBP/BPI1 peptides.

In order to assess the role of BgLBP/BPI1 *in vivo*, we undertook to decrease its expression by using RNA-interference mediated knock-down. Following double strand dsRNA injections, BgLBP/BPI1 protein abundance was analyzed by western blotting and showed a significant decrease in the albumen gland and in the egg masses after 12 and 18 days, respectively (Figure 6). The number of eggs per clutch collected over 28 days from parents treated with dsRNA of BgLBP/BPI1 was significantly lower than in the control experiment, whereby dsRNA of the luciferase gene was injected. Furthermore, the egg masses of the BgLBP/BPI1 dsRNA-treated snails showed a significant decrease in fecundity (Table 1). Thereby confirming that the albumen gland, the site of expression of BgLBP/BPI1, is directly involved in egg mass production [41]. After exposure to zoospores of *S. diclina*, eggs from parents silenced for BgLBP/BPI1 expression suffered an important decrease in their hatching rate, when compared to snails injected with control dsRNA (Table 1).

**Table 1:** BgLBP/BPI1 expression affects snail fecundity and egg survival to oomycete exposure.

	dsLUC injected snails (n = 30)	dsBgLBP/BPI1 injected snails (n = 30)
Number of egg masses laid per snail	5.0±1.4	5.5±1.4
Number of egg per egg mass	14.4±1.2	7.5±1.8**
% eggs hatching under control conditions (%)	64.6±5.1	57.4±12.2
% eggs hatching in the presence of *S. diclina*	49.1±4.9	25.7±8.3**

Fecundity was assessed as the number of egg masses and the number of eggs per egg mass laid during the first 28 days following the first injection of non-relevant luciferase dsRNA (dsLUC) or BgLBP/BPI1 dsRNA (dsBgLBP/BPI1). Egg hatching rate was assessed from egg masses collected from 12 to 21 days post-injection (period of optimum gene expression knock-down).

** Significantly different from values in dsLUC injected snails, P<0.01, (likelihood ratio test on nested models).

**Figure 7:**
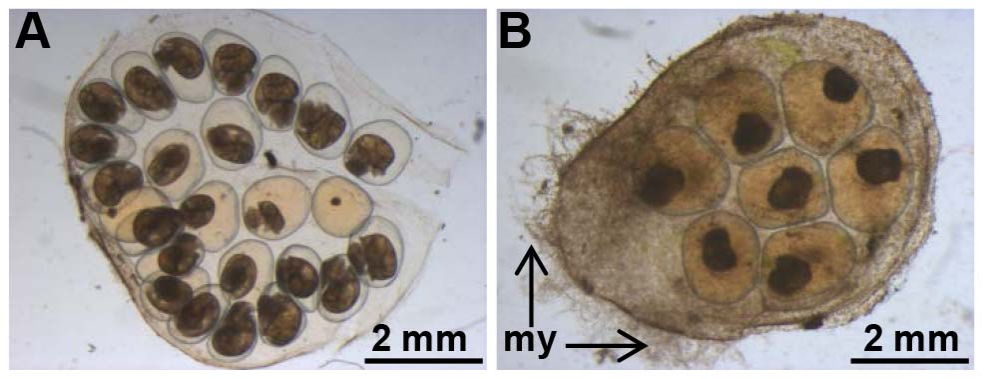
BgLBP/BPI1 is essential to protect eggs from oomycete infection. Typical egg mass from Luc- (A) or BgLBP/BPI1 (B) dsRNA-injected parents, after exposure to *S. diclina* zoospores. Note the well-developed mycelium (my). Egg masses from (A) and (B) have been laid 19–21 days after dsRNA injection to parents and have been exposed to zoospores at day 22. Observation was made after 8 days exposure to *S. diclina* zoospores.

The eggs from control-treated parents appeared healthy with a normal development of the embryos (Figure 7A), whereas the egg masses from parents treated with BgLBP/BPI1 dsRNA were covered by oomycete hyphae and the resulting infection impaired dramatically the survival of the snail embryos (Figure 7B).

## Discussion

Many invertebrate species lay fertilized eggs in nutritive egg masses that are highly suitable to the development of microorganisms [42], [43]. Although parental protection of eggs seems crucial to the survival of species, studies on the immune protection of invertebrate eggs are scarce. For example, an antibacterial activity was shown in eggs from 32 mollusk, 2 polychaete and 1 coral species, out of, respectively 34, 4 and 11 species tested [9], [42]. An antibacterial protein, the aplysianin-A was identified from eggs of the gastropod *Aplysia kurodai* [44], and an N-acetyl-galactosamine-binding lectin that agglutinates bacteria was identified in eggs from the pulmonate snail *Helix pomatia* [45]. Together with peptides of aplysianin, peptides of LBP/BPI proteins were identified in a proteomic study on *Biomphalaria glabrata* egg masses [15]. Here we characterized a *B. glabrata* LBP/BPI family member that is produced in the albumen gland and abundantly loaded into the egg masses. Consistent with the study on the BPI-like protein from *Crassostrea gigas* [27], we showed that the LPS and lipid A-binding activities against Gram-negative bacteria are conserved in BgLBP/BPI1. BgLBP/BPI1s did not exert any effect against the panel of microorganism tested. However, bacterial permeability activity was observed against *E. coli SBS363*, a mutant strain containing short-chain LPS. This is in agreement with previous studies reporting the resistance of Gram-negative bacteria harboring long lipopolysaccharide chain to the activity of hBPI [34], [46].

In addition to this expected anti-bacterial activity, we discovered a yet unsuspected anti-oomycete activity and demonstrated that BgLBP/BPI1 is a major fitness-related protein affecting both egg production under control conditions and offspring survival in the presence of pathogens. It is possible that the positive effect of BgLBP/BPI1 on the number of eggs produced is related to the glycoprotein nature of the molecule rather than to its antimicrobial activities. Interestingly, the glycoprotein HdAGP, identified from the snail *Helisoma duryi* albumen gland, was reported as the major nutritive glycoprotein secreted in the perivitelline fluid, and is also sharing sequence similarities with LBP/BPIs [43]. The content in BgLBP/BPI1 may therefore affect egg production as a major nutritive egg mass compound, independently of its antimicrobial action. However, once the egg masses are laid, we demonstrated that the biocidal activity of BgLBP/BPI1 affects offspring survival in the presence of oomycete pathogens.

LBP and BPIs are pleiotropic molecules, well characterized for their interactions with LPS from Gram-negative bacteria, but also reported to interact with other organisms such as Gram-positive bacteria and fungi [18], [47], [48]. A wide range of lipidic ligands have been reported for human LBP and BPI [18], [47], [48]. Our results further evidence the diversity of binding capabilities of LBP/BPIs as both BgLBP/BPI1 and hBPI can interact with an oomycete lipidic ligand that remains to be identified. Oomycetes do share physical characteristics with true fungi, including polarized hyphal extensions but they have a distinct evolutionary history and belong to the kingdom *Stramenopila*, which also includes brown algae and diatoms [49]. In contrast to fungi, they produce bi-flagellated swimming spores (zoospores) and the cell-wall of their cysts is composed of cellulose, β-glucans and hardly any chitin [49]. Oomycetes include some of the most devastating animal and plant pathogens. A few species cause Saprolegniosis in the aquaculture industry [38]. *Saprolegnia* species are endemic to freshwater habitat worldwide and are partly responsible for declining natural populations of salmonids and amphibians [50], [51]. Furthermore, the potato and tomato late-blight pathogen, *Phytophthora infestans* triggered the Irish Famine in the mid-1840s [37], [50], [52]. The potent oomycete killing activity of both BgLBP/BPI1 and hBPI was observed with three species belonging to two major oomycete orders, the Perenosporales and Saprolegniales [52]. Our observations demonstrate a conserved and broad-spectrum oomycete killing activity of BgLBP/BPI1, which may be of interest for both the agricultural and aquacultural sectors [53]. Interestingly, the specificity of this biocidal activity for the zoospore stage suggests that the ligand may be expressed specifically at this developmental stage. To date, despite the economic impact of oomycetes, there is no biochemical information on the membrane compounds of zoospores as studies have focused on identifying surface components of the cell wall of cysts [54], [55].

Collectively, our results significantly expand our knowledge of the multiple functions of LBP/BPI and highlight their importance in invertebrate biology. We demonstrated that LBP/BPI proteins display a conserved, potent and so far unexpected biocidal activity against zoospores from different oomycete orders. The precise binding and killing activity of the zoospores is unknown, but it is clear that BgLBP/BPI1 represents a major fitness-related protein transferred from parents to their clutches protecting snail eggs from widespread and lethal oomycete infections.

## Materials and Methods

### Characterization of BgLBP/BPI1

A partial cDNA sequence (EST GenBank accession number EB709540) was used to design specific primers and perform 5′- and 3′-RACE amplification (5′3′ RACE kit, 2^nd^ generation - Roche) according to the manufacturer's instructions. PCR products were cloned into pCR4-TOPO vector (Invitrogen) for sequencing. Sequence similarity searches were carried out using NCBI's BLAST-X program [56] against non-redundant databases with default parameters. Global sequence alignments were performed with Clustal W software [57]. The protein domains and signal peptide were predicted with the SMART [58] and SignalP [59] softwares, respectively.

### Animals and microorganisms

Adult *Biomphalaria glabrata* snails (albino strain) were raised in pond water and fed leaf lettuce *ad libitum* according to previously described procedures [60]. Bacterial and yeast strains used in this study were *Micrococcus luteus* (CIP A270), *Pseudomonas aeruginosa* (PA14) [61], *Bacillus cereus* (ATCC 11778), *Citrobacter freundii* (ATCC 8090), *Candida albicans* (a pathogenic strain isolated in patient no. 3 by Pr M. Koenig, CHU Strasbourg-Hautepierre) and *Saccharomyces cerevisiae* (Bioreference Laboratory – Institut Pasteur (Lille, France) as well as the *E. coli* SBS363, a Trp+ galU129 (truncated LPS) derivative of *E. coli* K12 strainD22 (gift from D. Destoumieux-Garzón, Université Montpellier 2). Bacterial strains were maintained in LB medium at 37°C and yeast in YPD medium at 28°C under standard conditions.

*S. mansoni* miracidia (swimming infective stage) were hatched from eggs axenically recovered from 50-days infected hamster livers according to previously described procedures [62]. Oomycete species used in the study were *Saprolegnia parasitica*, *S. diclina* and *Phytophthora parasitica*. *Saprolegnia* zoospores were obtained as described previously [63]. The average number of zoospores released was approximately 10^4^ zoospores per ml. *Phytophthora parasitica* was grown in 90 mm-diameter Petri dishes on 20% V8 agar media (350 ml V8 juice, 5 g CaCO_3_, 3.5 g agar) at 25°C for 6–8 days under continuous light. To induce zoospore release, *Phytophthora* isolates were placed at 4°C for 30 min. Mycelial cultures were then flooded with 10 ml of warm sterile water and left at 28°C for 30 min [40]. The average number of zoospores obtained was approximately 10^6^ zoospores per ml.

### Expression studies

Snail organs or tissues, namely albumen gland, hepatopancreas, headfoot, digestive tract and gonads were dissected under a binocular microscope, pooled from 10 individuals and frozen in liquid nitrogen. Snail circulating hemocytes were recovered from hemolymph collected prior to tissue dissections according to previously described procedures [62]. Total RNA and protein were simultaneously isolated using TRIZOL LS Reagent (Invitrogen) according to the manufacturer's instructions. Total RNA was quantified using a NanoDrop Spectrophotometer ND-1000 (Thermo Scientific). For cDNA synthesis, 50 ng of RNA from dissected tissues and hemocytes were used for reverse transcription using iScript cDNA Synthesis kit (Bio-Rad) and the oligo(dT) 20 primer.

The relative expression of BgLBP/BPI1 was monitored using Quantitative Real-time PCR on a DNA engine opticon 2 system (Biorad). Primers specific for *B. glabrata* ribosomal protein S19 (Genbank accession number CK988928) [64], elongation factor EEF1- α (Genbank accession number ES482381.1) and BgLBP/BPI1, were designed with primer 3 software and used for amplification in triplicate assays. The PCR cycling procedure was as follows: initial denaturation at 95°C for 10 min, followed by 40 cycles of amplification 95°C for 30 s, 60°C for 30 s and 68°C for 30 s for signal collection in each cycle. To assess the specificity of the PCR amplification, a melting curve analysis of the amplicon was performed at the end of each reaction and a single peak was always observed.

To examine the distribution of BgLBP/BPI1 protein in snail dissected tissues or egg masses, an anti-BgLBP/BPI1 antiserum was produced in a rabbit using the LAKAHIEKNRLIPDLLSYD and AQDKPGAVLRLNQEALDYGSR peptides and the polyclonal sera were purified using a peptide linked resin column (Proteogenix). Total protein contents of tissues were first determined by the BCA method (BC assay kit, Uptima) using albumin as a standard. 15 ug of tissue or egg mass proteins were loaded onto 10% SDS-PAGE gels and either silver stained using standard procedures, or transferred to a PVDF membrane (0.2 mm pore size) using a semi-dry blotting system. Western blots were performed using the custom anti-BgLBP/BPI1 antisera (Proteogenix).

### Production of recombinant and native BgLBP/BPI1 proteins

cDNA corresponding to the open-reading frame of BgLBP/BPI1 was ligated into the pMT/V5/His-A expression vector (Invitrogen). The *Drosophila* expression system with Schneider 2 (S2) cells (Invitrogen) was used to express recombinant C-terminally His-tagged full-length BgLBP/BPI1 (rBgLBP/BPI1) as described previously [65], [66]. Briefly, S2 cells were transiently transfected by calcium phosphate method with 1 µg of pMT/BgLBP/BPI1/V5/His-A vector and its expression was monitored by SDS-PAGE and western blotting after 3 days of induction with CuSO4 (500 mM). After confirmation of transient rBgLBP/BPI1 expression, stable cell lines were generated performing co-transfections along with 0.1 µg of PJL3 selection vector and 1 µg/ml puromycin. Establishment of stable cell lines and production of rBgLBP/BPI1 were carried out as described previously [66]. Nickel (II)-based immobilized metal affinity chromatography (Qiagen) in native conditions was performed to purify the recombinant BgLBP/BPI1 protein according to the manufacturer's protocol.

The native BgLBP/BPI1 protein (nBgLBP/BPI1) was purified from two-days old *B. glabrata* egg masses. Egg masses were homogenized in 20 mM acetate buffer, pH 4.5. The crude homogenate was centrifuged at 13000 rpm for 10 min to remove the gelatinous and solid debris. Supernatant containing nBgLBP/BPI1 was loaded onto SP Trisacryl M cation-exchange resin (BioSepra) equilibrated in 20 mM acetate buffer, pH 4.5. After washing 3 times with equilibration buffer, nBgLBP/BPI1 was eluted with 1 M NaCl, 20 mM acetate buffer, pH 4.5 and quantified by Bradford method. In order to analyze the sequence of the nBgLBP/BPI1, the purified protein was excised from a 10% SDS-PAGE gel and subjected to a MALDI TOF/TOF-MS analysis (Proteomic facility, University of Strasbourg, France). Protein identification was performed by subjecting the m/z values to Mascott software at an adjusted peptide mass tolerance of 50.000.000 ppm and/or 0.5 Da and at a fragment mass tolerance of 0.4 Da. For the subsequent activity assays, the purity of the purified nBgLBP/BPI1 protein was assessed after SDS-PAGE and silver staining. Assessments of both the egg mass volumes used for purification and the final concentration of the purified protein allowed to determine that the natural concentration of nBgLBP/BPI1 is in the range of 100 µg/ml of fresh 2 days old egg masses.

### LPS-binding activity

Binding of LPS or lipid A to rBgLBP/BPI1 and nBgLBP/BPI1 was assessed with a Biacore 3000 system (Biacore, GE Healthcare). RBgLBP/BPI1 and nBgLBP/BPI1 were immobilized at 7000 response units (RU) onto an activated CM5 sensor chip (Biacore) according to the manufacturer's instructions. Human BPI (hBPI - Athens Research, USA) and BSA proteins were immobilized using the same conditions as positive and negative control proteins, respectively. An activated and blocked flow-cell without immobilized ligand was used as a reference to evaluate nonspecific binding. HBS-EP running buffer (10 mM HEPES, 150 mM NaCl, 3 mM EDTA, and 0.005% Tween 20, pH 7.4) was used for sample dilution and analysis. Purified diphosphoryl lipid A from *E. coli* F583 Rd mutant and LPS from *E. coli* O26:B6 (Sigma) were sonicated 15 min at 25°C and injected at various concentrations. LPS was diluted at 50, 100, 250, 500, 1000 and 2000 nanograms and lipid A at 30, 60, 100, 150, 200, 300, 400, 500 nanograms and passed over the sensor chip at a flow rate of 50 µl/min. Regeneration was achieved with two washes of 20 mM NaOH for 5 min and 150 mM NaOH for 5 min for LPS and lipid A, respectively. Sensor chip was finally equilibrated with HBS-EP buffer for 2 min. All analyses were done at a constant temperature of 25°C. Data analysis was performed after subtraction of the uncoated flow-cell values by using BIAevaluation software version 4.1 (BIAcore). The association and dissociation phases of all sensor-grams were fitted globally. Kinetic parameters were then determined using a 1∶1 Langmuir binding model.

### Bacterial membrane permeability assays

The effect of proteins on the permeability of bacterial membranes was determined by flow cytometry using *E. coli SBS363* and the LIVE/DEAD BacLight Bacterial Viability Kit (Molecular probes). This kit enables assessment of bacterial viability based on membrane integrity by differentiating between bacteria with intact and damaged cytoplasmic membranes [67]. Bacterial culture in mid-logarithmic phase was adjusted to an optical density of A_600_ = 0.003 with poor-broth nutrient medium and treated with 10, 30, or 100 µg/ml of rBgLBP/BPI1 and nBgLBP/BPI1. BSA and hBPI were used at similar concentrations as a negative and positive control, respectively. Samples were incubated 1, 2 and 6 h at 28°C under vigorous shaking. Bacterial suspensions were stained with LIVE/DEAD BacLight staining reagent mixture (SYTO 9 and propidium iodide - PI) as described by the manufacturer. Staining was allowed for 5 min at room temperature in the dark. Flow cytometric measurements were performed on a FACSCanto II flow cytometer (BD Biosciences) with a 488 nm argon excitation laser. A total of 60,000 events were acquired and analyzed in each sample, using BD FACSDiVa software version 6.1.3 (BD Biosciences). Results are displayed as a percentage of permeabilized cells with respect to the negative control. The experiments were carried out three times independently.

### Assays for biocidal activity

The antibacterial activity was tested on *Micrococcus luteus*, *Bacillus cereus*, *Citrobacter freundii*, and *Pseudomonas aeruginosa* using the liquid growth inhibition method as previously described [68]. For antifungal activity assays, a similar liquid growth inhibition assay was performed using YPD medium. Microbial growth was controlled by measurement of the optical density at A_600_ after 6, 16 and 24 h incubation in proteins at 10, 50, 100 ug/ml. Fungal growth was additionally evaluated at 48 h. The percentage of growth (% growth) was deduced from the absorbance (OD) at 600 nm as previously described [69].

The effect on *S. mansoni* viability was tested on groups of 10–15 miracidia placed in 24-well plates. Miracidia were exposed to proteins at 10, 50, 100, 200 µg/ml at 28°C. Microscopic observations were performed at 30 min, 1, 2, 4, 6, 8 and 24 h of incubation.

The anti-oomycete assays were adjusted to the characteristics and life-cycles of the oomycete species. Zoospores of *S. parasitica* and *S. diclina* were adjusted to 10000 cells/ml and exposed to increasing concentrations (5, 10, 30, 100 µg/ml final concentration) of proteins. Because of the rapid encystment of zoospores into cysts, assessment of mortality was only performed at 30 min (prior to encystment of live zoospores). Zoospores of *Phytophthora parasitica* were adjusted to 500000 cells/ml and exposed to identical concentrations of proteins (5, 10, 30, 100 ug/ml final concentration). Microscopic observations of the number of live and dead zoospores were performed after 10, 30, 60, 120, 240 min of treatment. Zoospore-treated suspensions were stained immediately after incubation with the Live/Dead Cell Assay kit (Abcam) as described in the manufacturer's protocol. All conditions were tested in triplicate and assays were performed 3 times independently.

### RNA interference experiments

BgLBP/BPI1-specific primers containing a T7 promoter sequence were designed to amplify a 420-bp region of BgLBP/BPI1 used as a template for double stand RNA (dsRNA) synthesis according to manufacturer's instructions (MEGAScript T7 kit, Ambion). The firefly (*Photinus pyralis*) luciferase gene dsRNA (pGL3 vector, Promega) was produced and used as a non-relevant dsRNA control.

Each dsRNA (15 µg in 10 µl of sterile Chernin's Balanced Salt Solution - CBSS) was injected into the cardiac sinus of individual snails using a 10 µl Hamilton syringe with a 26 s needle [62]. A second injection was performed 12 days after the first dsRNA injection in order to optimize the knock-down efficiency. Groups of 10 snails were injected either with BgLBP/BPI1 or Luc dsRNA and were maintained under standard conditions. Egg masses produced during 28 days following the first dsRNA injection were scored, collected and observed under a stereoscopic microscope for assessment of the number of eggs in each egg mass. Egg masses laid from day 12 to 21 after the first dsRNA injection were either maintained under control conditions, or exposed to *Saprolegnia diclina* zoospores at a final concentration of 10^4^cells/ml. Egg masses were microscopically observed during the following 10 days to assess the egg hatching rate. Results are shown as the percentage of eggs hatched. RNA interference experiments were performed three times independently.

### Statistical analyses

All data were expressed as mean of three independent experiments plus or minus SE. Differences in relative BgLBP/BPI1 gene expression were tested for statistical significance by one-way ANOVA and the tukey-Kramer test (Software Prism v.5.0, GraphPad). Data from membrane-permeabilizing and antimicrobial (antibacterial, anti-oomycete) assays were analyzed by the chi-square test of independence [70] between treatments (rBgLBP/BPI1, nBgLBP/BPI1, hBPI and BSA) and the proportion of dead cells, using the computing environment R [71]. *Schistosoma mansoni* survival curves were analyzed by the Mantel-Cox log-rank test (Software Prism v.5.0, GraphPad). Results on snail fecundity and on egg viability were statistically analyzed by the likelihood ratio test between nested models [71]. Briefly, three variables were considered in these models; the mean number of egg per snail, the mean number of egg masses per snail and the mean number of eggs per egg mass. To test the effect of the BgLBP/BPI1 silencing on snail fecundity for each of the three variables, linear mixed models were fitted with the function lmr (LML 4 package of R software). In each case the model contains the BgLBP/BPI1 silencing as fixed effect and the time and the replicates as random effects. To normalize the data the mean number of eggs and egg mass per snail were log transformed. To assess the effect of the oomycete infection on the hatching rate of BgLBP/BPI1 silenced eggs, saturated binomial generalized linear mixed models were fitted. This model contained as fixed effects the BgLBP/BPI1 silencing, the oomycete infection, the time and all interactions among these variables; and as random effects, the replicates. To account for over-dispersion, individual level of variability was added. From this model variable selection of fixed effect was based on the AICc (dredge function of MuMIn package of R software) then the selected fixed effect was analyzed by the likelihood ratio test. A P value of <0.05 was considered statistically significant. Where indicated in figures: *P<0.05, **P<0.01, ***P<0.001.

## Supporting Information

Figure S1 **BgLBP/BPI1 is a member of the LBP/BPI family.** Alignment of the translated BgLBP/BPI1 sequence with Human LBP (HsLBP, AAA59493.1), Human BPI (HsBPI, AAA51841.1), *Crassostrea gigas* BPI1 (CgBPI1, AAN84552.1), *C. gigas* BPI2 (CgBPI2, ADN05759.1) and *Helisoma duryi* Albumen Gland Protein (HdAGP, Mukai 2004). Alignments were performed with ClustalW. Conserved amino acids are indicated by asterisks. The putative cleavage site of the signal peptide is indicated by an arrow. The N- and C- terminal domains are highlighted in gray. The LPS binding domain is boxed. The proline-rich domain is boxed with a dashed line. Cysteins forming the disulfide bond conserved in the LBP/BPI family are linked by a line. Peptides from the native egg mass protein recorded by mass spectrometry are highlighted in blue.
(TIF)

Figure S2 **BgLBP/BPI1 and hBPI do not show significant effect on the viability of** ***Schistosoma mansoni*** **miracidia.** Parasite survival after exposure to rBgLBP/BPI1 (red), hBPI (blue) or BSA (green) at 100 µg/ml. Negative control (black) consists in miracidia without treatment. Results are mean percentages of three independent experiments (Mantel-Cox log-rank test, Software Prism v.5.0, GraphPad).
(TIF)

Figure S3 **rBgLBP/BPI1 does not show a significant effect on the viability of various micro-organisms.** Survival rate of various Gram-positive, gram-negative bacteria or fungi after exposure to rBgLBP/BPI1 (A) or hBPI (B) at 100 µg/ml. Results are mean percentages (± SE) of three independent experiments.
(TIF)

Figure S4 **BgLBP/BPI1s and hBPI display anti-oomycete activity against** ***P. parasitica*** **zoospores.** (A) *P. parasitica* developmental stages, namely (a) zoospores, (b) cysts and (c) microcolonies. (B) Representative pictures of the anti-oomycete effect of (a) BSA, (b) hBPI, (c) rBgLBP/BPI1 and (d) nBgLBP/BPI1 at 30 µg/ml on *P. parasitica* zoospores. Pictures were taken after 30 min of incubation. Scale bars represent 20 µm.
(TIF)

## References

[1] Flajnik MF, Du Pasquier L (2004) Evolution of innate and adaptive immunity: can we draw a line? Trends Immunol 25: 640–644. 15530832 10.1016/j.it.2004.10.001

[2] Hasselquist D, Nilsson JA (2009) Maternal transfer of antibodies in vertebrates: trans-generational effects on offspring immunity. Philos Trans R Soc Lond B Biol Sci 364: 51–60. 18926976 10.1098/rstb.2008.0137 PMC2666691

[3] Chucri TM, Monteiro JM, Lima AR, Salvadori ML, Kfoury JR Jr, et al (2010) A review of immune transfer by the placenta. J Reprod Immunol 87: 14–20. 20956021 10.1016/j.jri.2010.08.062

[4] Swain P, Nayak SK (2009) Role of maternally derived immunity in fish. Fish Shellfish Immunol 27: 89–99. 19442742 10.1016/j.fsi.2009.04.008

[5] Haine ER (2008) Symbiont-mediated protection. Proc Biol Sci 275: 353–361. 18055391 10.1098/rspb.2007.1211 PMC2213712

[6] Jaenike J, Unckless R, Cockburn SN, Boelio LM, Perlman SJ (2010) Adaptation via symbiosis: recent spread of a *Drosophila* defensive symbiont. Science 329: 212–215. 20616278 10.1126/science.1188235

[7] Hoffmann JA, Reichhart JM (2002) *Drosophila* innate immunity: an evolutionary perspective. Nat Immunol 3: 121–126. 11812988 10.1038/ni0202-121

[8] Dishaw LJ, Litman GW (2009) Invertebrate allorecognition: the origins of histocompatibility. Curr Biol 19: R286–288. 19368870 10.1016/j.cub.2009.02.035 PMC3699862

[9] Marquis CP, Baird AH, de Nys R, Holmström C, Koziumi N (2005) An evaluation of the antimicrobial properties of the eggs of 11 species of scleractinian corals. Coral Reefs 24: 248–253.

[10] Liang Y, Zhang S, Wang Z (2009) Alternative complement activity in the egg cytosol of amphioxus *Branchiostoma belcheri*: evidence for the defense role of maternal complement components. PLoS One 4: e4234. 19156196 10.1371/journal.pone.0004234 PMC2617767

[11] Fraune S, Augustin R, Bosch TC (2011) Embryo protection in contemporary immunology: Why bacteria matter. Commun Integr Biol 4: 369–372. 21966549 10.4161/cib.4.4.15159 PMC3181499

[12] Groombridge B, Jenkins MD (2002) World Atlas of Biodiversity: earth's living resources in the 21st century: University of California Press, Berkeley, USA. 360 p.

[13] Gryseels B, Polman K, Clerinx J, Kestens L (2006) Human schistosomiasis. Lancet 368: 1106–1118. 16997665 10.1016/S0140-6736(06)69440-3

[14] Pimentel D (1957) Life History of *Australorbis glabratus*, The Intermediate Snail Host of *Schistosoma mansoni* in Puerto Rico. Ecology 38: 576–580. 13435434

[15] Hathaway JJ, Adema CM, Stout BA, Mobarak CD, Loker ES (2010) Identification of protein components of egg masses indicates parental investment in immunoprotection of offspring by *Biomphalaria glabrata* (gastropoda, mollusca). Dev Comp Immunol 34: 425–435. 19995576 10.1016/j.dci.2009.12.001 PMC2813990

[16] Bingle CD, Craven CJ (2004) Meet the relatives: a family of BPI- and LBP-related proteins. Trends Immunol 25: 53–55. 15106612 10.1016/j.it.2003.11.007

[17] Beamer LJ, Fischer D, Eisenberg D (1998a) Detecting distant relatives of mammalian LPS-binding and lipid transport proteins. Protein Sci 7: 1643–1646. 9684900 10.1002/pro.5560070721 PMC2144061

[18] Canny G, Levy O (2008) Bactericidal/permeability-increasing protein (BPI) and BPI homologs at mucosal sites. Trends Immunol 29: 541–547. 18838299 10.1016/j.it.2008.07.012

[19] Elsbach P, Weiss J (1993) The bactericidal/permeability-increasing protein (BPI), a potent element in host-defense against gram-negative bacteria and lipopolysaccharide. Immunobiology 187: 417–429. 8330906 10.1016/S0171-2985(11)80354-2

[20] Fenton MJ, Golenbock DT (1998) LPS-binding proteins and receptors. J Leukoc Biol 64: 25–32. 9665271 10.1002/jlb.64.1.25

[21] Elsbach P, Weiss J (1998) Role of the bactericidal/permeability-increasing protein in host defence. Curr Opin Immunol 10: 45–49. 9523110 10.1016/s0952-7915(98)80030-7

[22] Schultz H, Hume J, Zhang de S, Gioannini TL, Weiss JP (2007) A novel role for the bactericidal/permeability increasing protein in interactions of gram-negative bacterial outer membrane blebs with dendritic cells. J Immunol 179: 2477–2484. 17675509 10.4049/jimmunol.179.4.2477

[23] Weiss J (2003) Bactericidal/permeability-increasing protein (BPI) and lipopolysaccharide-binding protein (LBP): structure, function and regulation in host defence against Gram-negative bacteria. Biochem Soc Trans 31: 785–790. 12887306 10.1042/bst0310785

[24] Marra MN, Wilde CG, Collins MS, Snable JL, Thornton MB, et al (1992) The role of bactericidal/permeability-increasing protein as a natural inhibitor of bacterial endotoxin. J Immunol 148: 532–537. 1729370

[25] Schumann RR, Leong SR, Flaggs GW, Gray PW, Wright SD, et al (1990) Structure and function of lipopolysaccharide binding protein. Science 249: 1429–1431. 2402637 10.1126/science.2402637

[26] Altincicek B, Vilcinskas A (2007) Analysis of the immune-related transcriptome of a lophotrochozoan model, the marine annelid *Platynereis dumerilii* . Front Zool 4: 18. 17617895 10.1186/1742-9994-4-18 PMC1939704

[27] Gonzalez M, Gueguen Y, Destoumieux-Garzon D, Romestand B, Fievet J, et al (2007) Evidence of a bactericidal permeability increasing protein in an invertebrate, the *Crassostrea gigas* Cg-BPI. Proc Natl Acad Sci U S A 104: 17759–17764. 17965238 10.1073/pnas.0702281104 PMC2077063

[28] Krasity BC, Troll JV, Weiss JP, McFall-Ngai MJ (2011) LBP/BPI proteins and their relatives: conservation over evolution and roles in mutualism. Biochem Soc Trans 39: 1039–1044. 21787344 10.1042/BST0391039 PMC3679904

[29] Guillou F, Mitta G, Galinier R, Coustau C (2007) Identification and expression of gene transcripts generated during an anti-parasitic response in *Biomphalaria glabrata* . Dev Comp Immunol 31: 657–671. 17166585 10.1016/j.dci.2006.10.001

[30] de Jong-Brink M, Goldschmeding J (1983) Endocrine and nervous regulation of female reproductive activity in the gonad and albumen gland of *Lymnaea stagnalis*. In: Lever JB, HH., editor. Molluscan Neuroendocrinology. Amsterdam: North Holland Press pp. 126–131.

[31] Miller AN, Ofori K, Lewis F, Knight M (1996) *Schistosoma mansoni*: use of a subtractive cloning strategy to search for RFLPs in parasite-resistant *Biomphalaria glabrata* . Exp Parasitol 84: 420–428. 8948331 10.1006/expr.1996.0130

[32] Beamer LJ, Carroll SF, Eisenberg D (1998b) The BPI/LBP family of proteins: a structural analysis of conserved regions. Protein Sci 7: 906–914. 9568897 10.1002/pro.5560070408 PMC2143972

[33] Tan NS, Ho B, Ding JL (2000) High-affinity LPS binding domain(s) in recombinant factor C of a horseshoe crab neutralizes LPS-induced lethality. FASEB J 14: 859–870. 10783139 10.1096/fasebj.14.7.859

[34] Capodici C, Chen S, Sidorczyk Z, Elsbach P, Weiss J (1994) Effect of lipopolysaccharide (LPS) chain length on interactions of bactericidal/permeability-increasing protein and its bioactive 23-kilodalton NH2-terminal fragment with isolated LPS and intact *Proteus mirabilis* and *Escherichia coli* . Infect Immun 62: 259–265. 8262637 10.1128/iai.62.1.259-265.1994 PMC186095

[35] Gazzano-Santoro H, Parent JB, Grinna L, Horwitz A, Parsons T, et al (1992) High-affinity binding of the bactericidal/permeability-increasing protein and a recombinant amino-terminal fragment to the lipid A region of lipopolysaccharide. Infect Immun 60: 4754–4761. 1398985 10.1128/iai.60.11.4754-4761.1992 PMC258228

[36] Lamping N, Hoess A, Yu B, Park TC, Kirschning CJ, et al (1996) Effects of site-directed mutagenesis of basic residues (Arg 94, Lys 95, Lys 99) of lipopolysaccharide (LPS)-binding protein on binding and transfer of LPS and subsequent immune cell activation. J Immunol 157: 4648–4656. 8906845

[37] Kamoun S (2003) Molecular genetics of pathogenic oomycetes. Eukaryot Cell 2: 191–199. 12684368 10.1128/EC.2.2.191-199.2003 PMC154851

[38] Phillips AJ, Anderson VL, Robertson EJ, Secombes CJ, van West P (2008) New insights into animal pathogenic oomycetes. Trends Microbiol 16: 13–19. 18096392 10.1016/j.tim.2007.10.013

[39] Balakrishnan A, Marathe SA, Joglekar M, Chakravortty D (2012) Bactericidal/permeability increasing protein: A multifaceted protein with functions beyond LPS neutralization. Innate Immun 19 4: 339–47. 23160386 10.1177/1753425912465098

[40] Galiana E, Fourre S, Engler G (2008) *Phytophthora parasitica* biofilm formation: installation and organization of microcolonies on the surface of a host plant. Environ Microbiol 10: 2164–2171. 18430009 10.1111/j.1462-2920.2008.01619.x

[41] Bai G, Li J, Christensen BM, Yoshino TP (1996) Phenoloxidase activity in the reproductive system and egg masses of the pulmonate gastropod, *Biomphalaria glabrata* . Comp Biochem Physiol B Biochem Mol Biol 114: 353–359. 8840512 10.1016/0305-0491(96)00045-4

[42] Benkendorff K, Davis AR, Bremner JB (2001) Chemical defense in the egg masses of benthic invertebrates: an assessment of antibacterial activity in 39 mollusks and 4 polychaetes. J Invertebr Pathol 78: 109–118. 11812113 10.1006/jipa.2001.5047

[43] Mukai ST, Hoque T, Morishita F, Saleuddin ASM (2004) Cloning and Characterization of a Candidate Nutritive Glycoprotein from the Albumen Gland of the Freshwater Snail, *Helisoma duryi* (Mollusca: Pulmonata). Invertebrate Biology 123: 83–92.

[44] Takamatsu N, Shiba T, Muramoto K, Kamiya H (1995) Molecular cloning of the defense factor in the albumen gland of the sea hare *Aplysia kurodai* . FEBS Lett 377: 373–376. 8549758 10.1016/0014-5793(95)01375-X

[45] Sanchez JF, Lescar J, Chazalet V, Audfray A, Gagnon J, et al (2006) Biochemical and structural analysis of *Helix pomatia* agglutinin. A hexameric lectin with a novel fold. J Biol Chem 281: 20171–20180. 16704980 10.1074/jbc.M603452200

[46] Weiss J, Beckerdite-Quagliata S, Elsbach P (1980) Resistance of gram-negative bacteria to purified bactericidal leukocyte proteins: relation to binding and bacterial lipopolysaccharide structure. J Clin Invest 65: 619–628. 6986410 10.1172/JCI109707 PMC371403

[47] Schroder NW, Schumann RR (2005) Non-LPS targets and actions of LPS binding protein (LBP). J Endotoxin Res 11: 237–242. 16176661 10.1179/096805105X37420

[48] Zweigner J, Gramm HJ, Singer OC, Wegscheider K, Schumann RR (2001) High concentrations of lipopolysaccharide-binding protein in serum of patients with severe sepsis or septic shock inhibit the lipopolysaccharide response in human monocytes. Blood 98: 3800–3808. 11739189 10.1182/blood.v98.13.3800

[49] Beakes GW, Glockling SL, Sekimoto S (2012) The evolutionary phylogeny of the oomycete “fungi”. Protoplasma 249: 3–19. 21424613 10.1007/s00709-011-0269-2

[50] van West P (2006) *Saprolegnia parasitica*, an oomycete pathogen with a fishy appetite: new challenges for an old problem. Mycologist 20: 99–104.

[51] Romansic JM, Diez KA, Higashi EM, Johnson JE, Blaustein AR (2009) Effects of the pathogenic water mold *Saprolegnia ferax* on survival of amphibian larvae. Dis Aquat Organ 83: 187–193. 19402452 10.3354/dao02007

[52] Judelson HS (2012) Dynamics and innovations within oomycete genomes: insights into biology, pathology, and evolution. Eukaryot Cell 11: 1304–1312. 22923046 10.1128/EC.00155-12 PMC3486027

[53] Baron O, Reichhart JM, Ponchet M, Coustau C (2013) Anti-oomycete activity fo lipopolysaccharide (LPS)-binding proteins/bactericidal/permeability-increasing proteins. WO/2013/120619. In: INRA/CNRS, editor. WIPO Patentscope. France.

[54] Tyler BM (2009) Entering and breaking: virulence effector proteins of oomycete plant pathogens. Cell Microbiol 11: 13–20. 18783481 10.1111/j.1462-5822.2008.01240.x

[55] Melida H, Sandoval-Sierra JV, Dieguez-Uribeondo J, Bulone V (2013) Analyses of extracellular carbohydrates in oomycetes unveil the existence of three different cell wall types. Eukaryot Cell 12: 194–203. 23204192 10.1128/EC.00288-12 PMC3571302

[56] Altschul SF, Madden TL, Schaffer AA, Zhang J, Zhang Z, et al (1997) Gapped BLAST and PSI-BLAST: a new generation of protein database search programs. Nucleic Acids Res 25: 3389–3402. 9254694 10.1093/nar/25.17.3389 PMC146917

[57] Larkin MA, Blackshields G, Brown NP, Chenna R, McGettigan PA, et al (2007) Clustal W and Clustal X version 2.0. Bioinformatics 23: 2947–2948. 17846036 10.1093/bioinformatics/btm404

[58] Schultz J, Copley RR, Doerks T, Ponting CP, Bork P (2000) SMART: a web-based tool for the study of genetically mobile domains. Nucleic Acids Res 28: 231–234. 10592234 10.1093/nar/28.1.231 PMC102444

[59] Nielsen H, Krogh A (1998) Prediction of signal peptides and signal anchors by a hidden Markov model. Proc Int Conf Intell Syst Mol Biol 6: 122–130. 9783217

[60] Guillou F, Mitta G, Dissous C, Pierce R, Coustau C (2004) Use of individual polymorphism to validate potential functional markers: case of a candidate lectin (BgSel) differentially expressed in susceptible and resistant strains of *Biomphalaria glabrata* . Comp Biochem Physiol B Biochem Mol Biol 138: 175–181. 15193273 10.1016/j.cbpc.2004.03.010

[61] Limmer S, Haller S, Drenkard E, Lee J, Yu S, et al (2011) *Pseudomonas aeruginosa* RhlR is required to neutralize the cellular immune response in a *Drosophila melanogaster* oral infection model. Proc Natl Acad Sci U S A 108: 17378–17383. 21987808 10.1073/pnas.1114907108 PMC3198323

[62] Baeza Garcia A, Pierce RJ, Gourbal B, Werkmeister E, Colinet D, et al (2010) Involvement of the cytokine MIF in the snail host immune response to the parasite *Schistosoma mansoni* . PLoS Pathog 6: e1001115. 20886098 10.1371/journal.ppat.1001115 PMC2944803

[63] van West P, de Bruijn I, Minor KL, Phillips AJ, Robertson EJ, et al (2010) The putative RxLR effector protein SpHtp1 from the fish pathogenic oomycete *Saprolegnia parasitica* is translocated into fish cells. FEMS Microbiol Lett 310: 127–137. 20659163 10.1111/j.1574-6968.2010.02055.x

[64] Mitta G, Galinier R, Tisseyre P, Allienne JF, Girerd-Chambaz Y, et al (2005) Gene discovery and expression analysis of immune-relevant genes from *Biomphalaria glabrata* hemocytes. Dev Comp Immunol 29: 393–407. 15707661 10.1016/j.dci.2004.10.002

[65] Tauszig S, Jouanguy E, Hoffmann JA, Imler J-L (2000) Toll-related receptors and the control of antimicrobial peptide expression in *Drosophila* . Proceedings of the National Academy of Sciences 97: 10520–10525. 10.1073/pnas.180130797 PMC27057 10973475

[66] Gardsvoll H, Hansen LV, Jorgensen TJ, Ploug M (2007) A new tagging system for production of recombinant proteins in *Drosophila* S2 cells using the third domain of the urokinase receptor. Protein Expr Purif 52: 384–394. 17215141 10.1016/j.pep.2006.11.013

[67] Berney M, Hammes F, Bosshard F, Weilenmann HU, Egli T (2007) Assessment and interpretation of bacterial viability by using the LIVE/DEAD BacLight Kit in combination with flow cytometry. Appl Environ Microbiol 73: 3283–3290. 17384309 10.1128/AEM.02750-06 PMC1907116

[68] Hetru C, Bulet P (1997) Strategies for the isolation and characterization of antimicrobial peptides of invertebrates. Methods Mol Biol 78: 35–49. 9276295 10.1385/0-89603-408-9:35

[69] Barbault F, Landon C, Guenneugues M, Meyer JP, Schott V, et al (2003) Solution structure of Alo-3: a new knottin-type antifungal peptide from the insect *Acrocinus longimanus* . Biochemistry 42: 14434–14442. 14661954 10.1021/bi035400o

[70] Hope ACA (1968) A Simplified Monte Carlo Significance Test Procedure. Journal of the Royal Statistical Society Series B (Methodological) 30: 582–598.

[71] Team RDC (2005) R: A language and environment for statistical computing. In: Computing FfS, editor. 2.11.1 ed. Vienna, Austria.

