# Supplementary figures and images for: Parental Transfer of the Antimicrobial Protein LBP/BPI Protects Biomphalaria glabrata Eggs against Oomycete Infections
Source: PLoS Pathog. 2013 Dec 19;9(12):e1003792. doi: 10.1371/journal.ppat.1003792 (PMC3868517; doi:10.1371/journal.ppat.1003792)

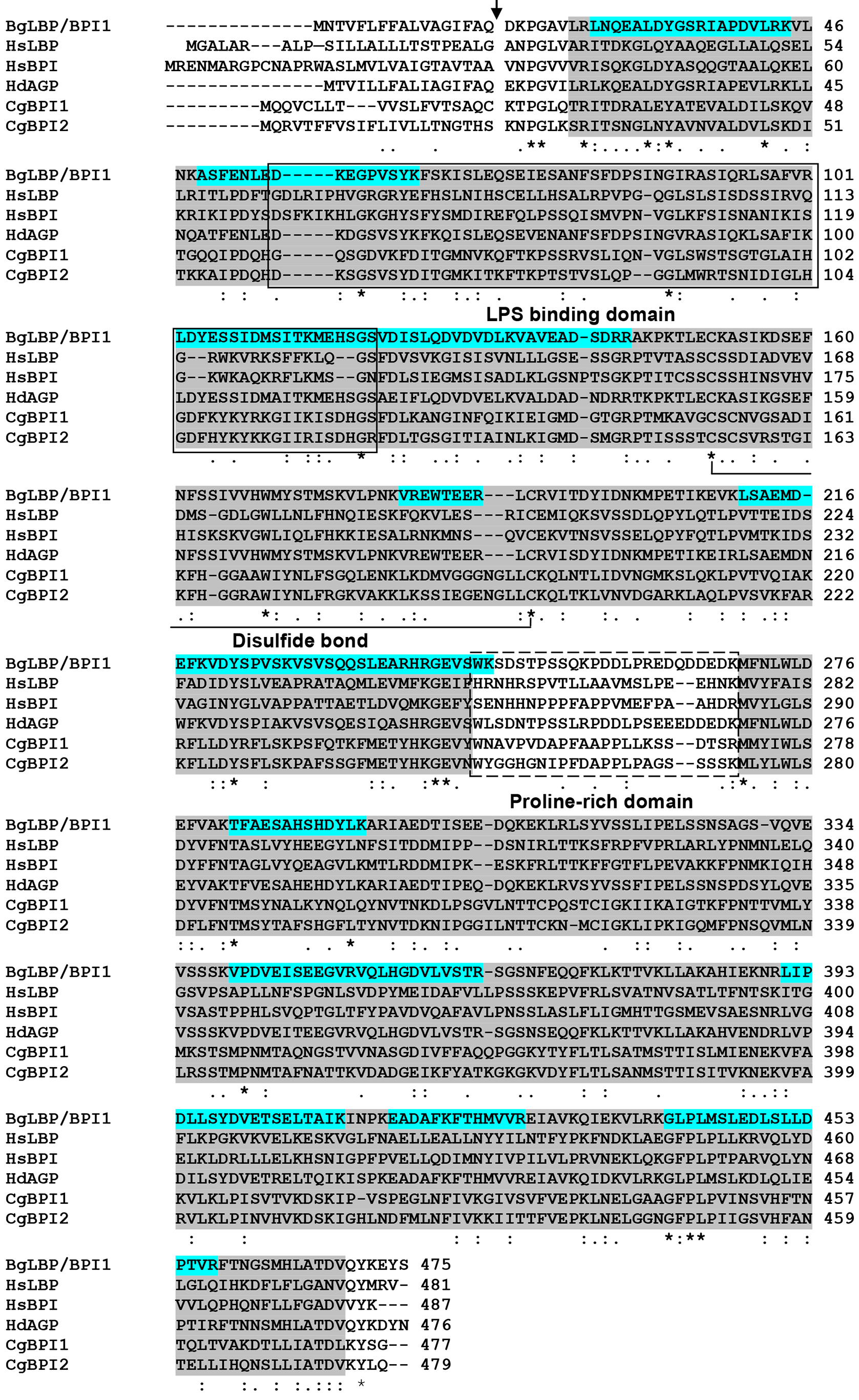

Supplement: Figure S1 — BgLBP/BPI1 is a member of the LBP/BPI family. Alignment of the translated BgLBP/BPI1 sequence with Human LBP (HsLBP, AAA59493.1), Human BPI (HsBPI, AAA51841.1), Crassostrea gigas BPI1 (CgBPI1, AAN84552.1), C. gigas BPI2 (CgBPI2, ADN05759.1) and Helisoma duryi Albumen Gland Protein (HdAGP, Mukai 2004). Alignments were performed with ClustalW. Conserved amino acids are indicated by asterisks. The putative cleavage site of the signal peptide is indicated by an arrow. The N- and C- terminal domains are highlighted in gray. The LPS binding domain is boxed. The proline-rich domain is boxed with a dashed line. Cysteins forming the disulfide bond conserved in the LBP/BPI family are linked by a line. Peptides from the native egg mass protein recorded by mass spectrometry are highlighted in blue. (TIF) [file ppat.1003792.s001.tif]

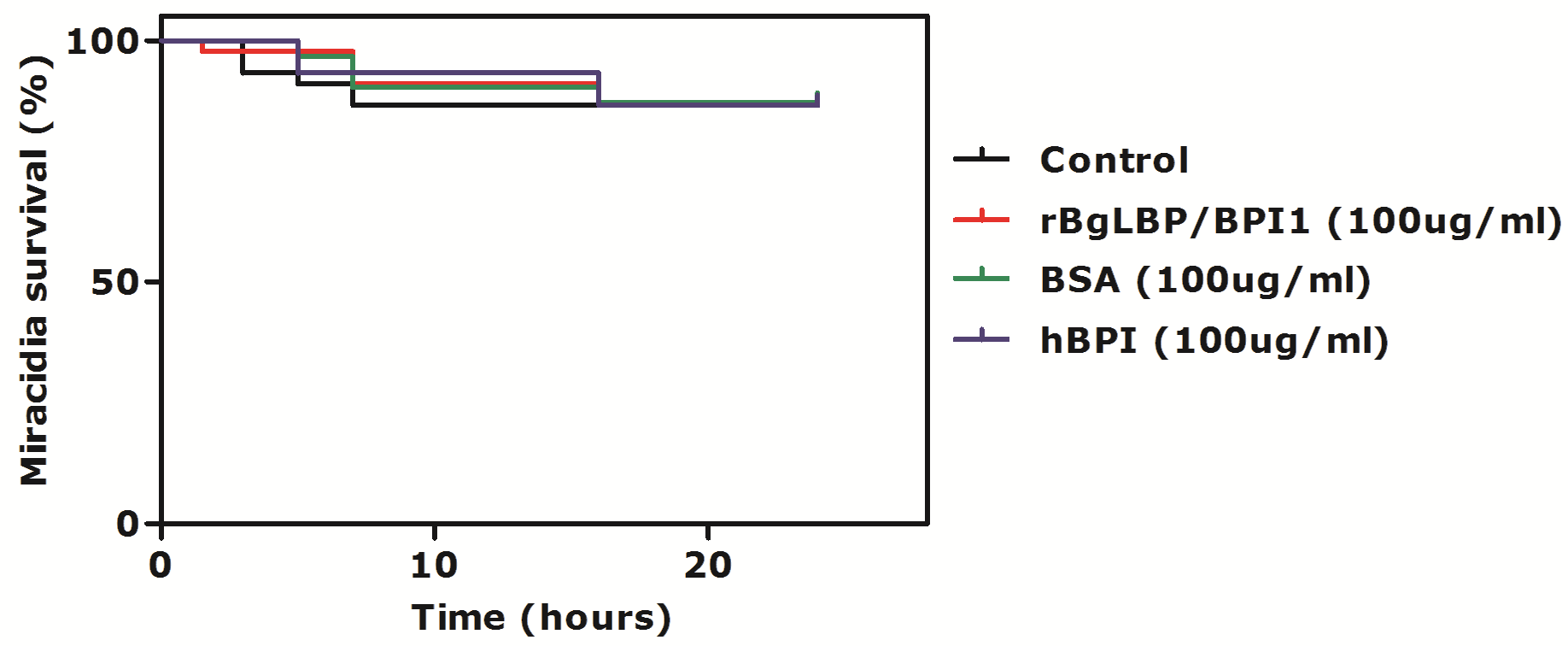

Supplement: Figure S2 — BgLBP/BPI1 and hBPI do not show significant effect on the viability of Schistosoma mansoni miracidia. Parasite survival after exposure to rBgLBP/BPI1 (red), hBPI (blue) or BSA (green) at 100 µg/ml. Negative control (black) consists in miracidia without treatment. Results are mean percentages of three independent experiments (Mantel-Cox log-rank test, Software Prism v.5.0, GraphPad). (TIF) [file ppat.1003792.s002.tif]

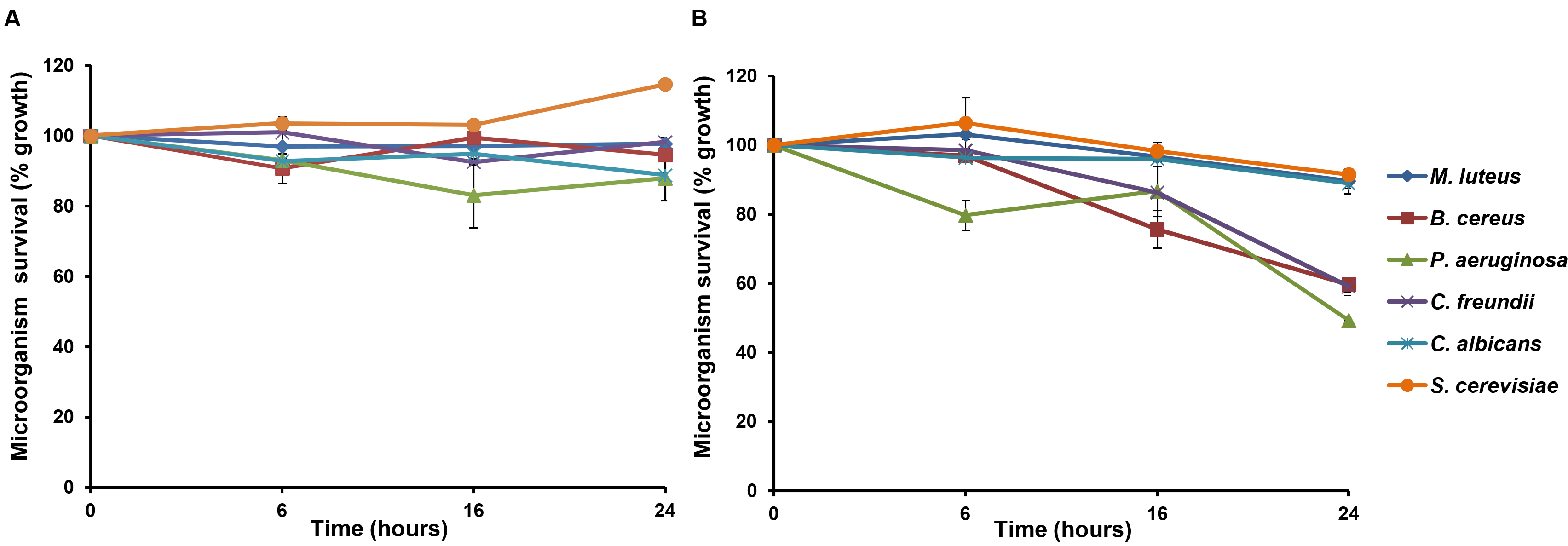

Supplement: Figure S3 — rBgLBP/BPI1 does not show a significant effect on the viability of various micro-organisms. Survival rate of various Gram-positive, gram-negative bacteria or fungi after exposure to rBgLBP/BPI1 (A) or hBPI (B) at 100 µg/ml. Results are mean percentages (± SE) of three independent experiments. (TIF) [file ppat.1003792.s003.tif]

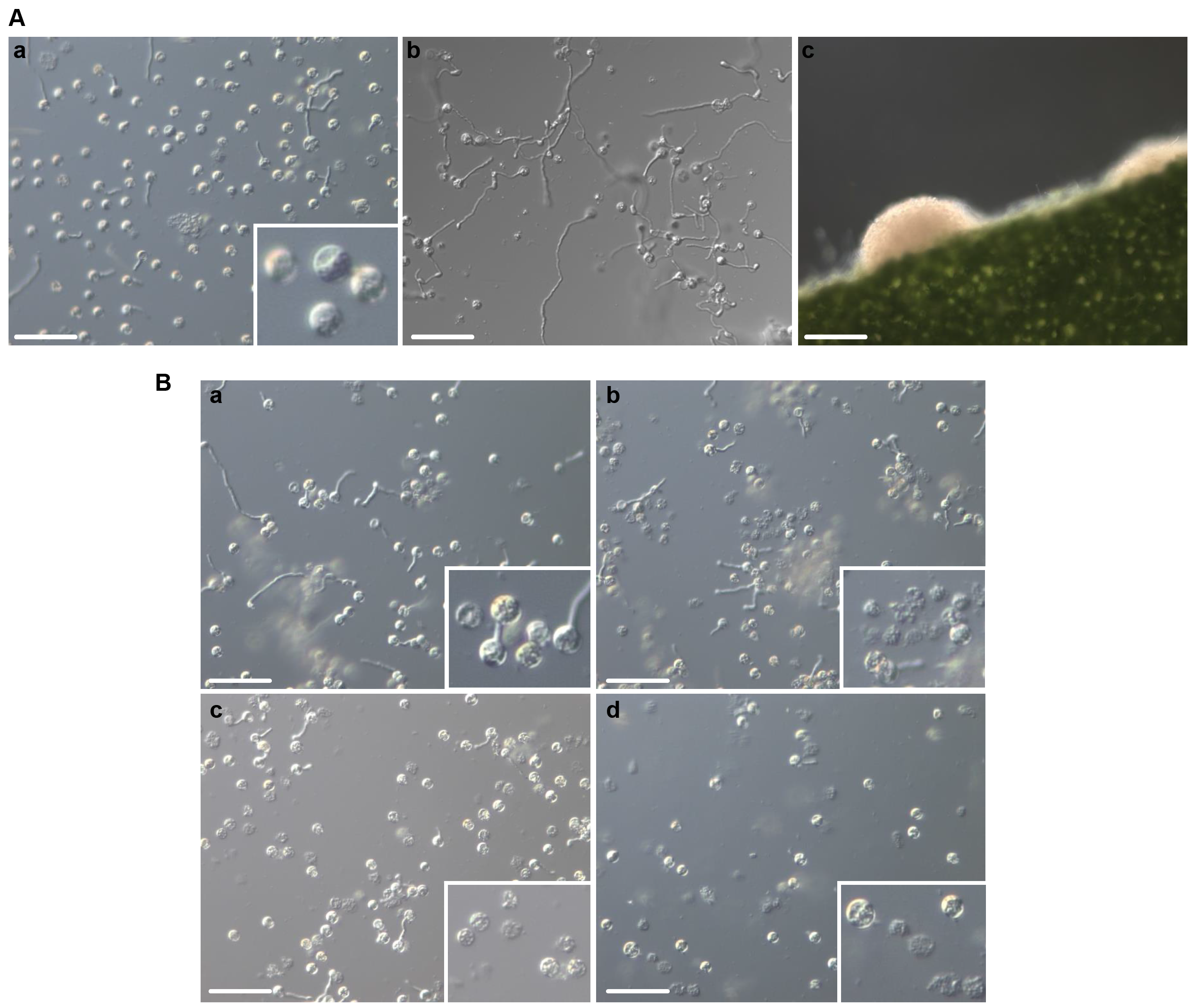

Supplement: Figure S4 — BgLBP/BPI1s and hBPI display anti-oomycete activity against P. parasitica zoospores. (A) P. parasitica developmental stages, namely (a) zoospores, (b) cysts and (c) microcolonies. (B) Representative pictures of the anti-oomycete effect of (a) BSA, (b) hBPI, (c) rBgLBP/BPI1 and (d) nBgLBP/BPI1 at 30 µg/ml on P. parasitica zoospores. Pictures were taken after 30 min of incubation. Scale bars represent 20 µm. (TIF) [file ppat.1003792.s004.tif]
